# Third exposure to COVID-19 infection or vaccination differentially impacts T cell responses

**DOI:** 10.1016/j.jinf.2025.106598

**Published:** 2025-08-21

**Authors:** Gift Ahimbisibwe, David Greenwood, Katalin Andrea Wilkinson, Joshua Gahir, Hermaleigh Townsley, Murad Miah, Philip Bawumia, Charlotte Chaloner, Dina Levi, Philip Hobson, Andy Riddell, Agnieszka Hobbs, Giulia Dowgier, Rebecca Penn, Theo Sanderson, Phoebe Stevenson-Leggett, Odiesia Daley, James Bazire, Ruth Harvey, Ashley S. Fowler, Callie Smith, Mauro Miranda, Nicola O’Reilly, Scott Warchal, Karen Ambrose, Amy Strange, Gavin Kelly, Svend Kjar, Legacy Investigators, Richard Gilson, Rupert Beale, George Kassiotis, Tumena Corrah, Padmasayee Papineni, Bryan Williams, Vincenzo Libri, Steve Gamblin, Sonia Gandhi, Charles Swanton, David LV Bauer, Robert John Wilkinson, Edward J. Carr, Emma C. Wall

**Affiliations:** a The Francis Crick Institute, NW1 1AT London, UK; b COVID Surveillance Unit, The Francis Crick Institute, 1 Midland Road, London NW1 1AT, UK; c Worldwide Influenza Centre, The Francis Crick Institute, 1 Midland Road, London NW1 1AT, UK; d Welcome Discovery Research Platforms in Infection, Centre for Infectious Diseases Research in Africa (CIDRI-Africa), Institute of Infectious Disease and Molecular Medicine and Department of Medicine, University of Cape Town, Cape Town 7925, Republic of South Africa; e National Institute for Health Research (NIHR) University College London Hospitals (UCLH) Biomedical Research Centre and NIHR UCLH Clinical Research Facility, UK; f University College London, Gower Street, London, Uk; g Genotype-to-Phenotype 2 Consortium (G2P2-UK), UK; h Department of Infectious Diseases, Imperial College London, W12 0NN London, UK; i Research Department of Infection, Division of Infection and Immunity, University College London, UK; j King’s College London, St Thomas’ Campus, Westminster Bridge Road, London, UK

**Keywords:** SARS-CoV-2, Vaccination, T cells, Immunity, Infection exposure

## Abstract

**Background:**

In 2021, the rapid rollout of two doses of SARS-CoV-2 vaccines reduced COVID-19 severity and mortality. However, further vaccine doses as a prime-boost schedule were limited, and lifting of public health restrictions by late 2021 frequently led to infection, rather than vaccine, as a third exposure.

**Objective:**

To compare how the third exposure through mRNA booster or SARS-CoV-2 infection shapes humoral and cellular immunity following two vaccine doses.

**Methods:**

We compared immune responses after the third exposure in healthy adults enrolled in the UCLH-Crick Legacy cohort study (NCT04750356) between those receiving ancestral spike-encoded mRNA booster (vaccine immunity, n = 38) or COVID-19 infection (hybrid immunity, n = 13) following two vaccine doses. Immune profiles were evaluated using live virus neutralization assays, IFN-γ ELISpot, Luminex assay, flow cytometry and mass cytometry.

**Results:**

Both total anti-Spike IgG and variant-specific neutralising antibodies were comparable following infection or vaccine as a third exposure. Overall, T cell populations were similar but functionally different. CD8^+^ Effector Memory (TEM) cells in the vaccine group showed higher expression of CD69 and Granzyme B following stimulation with SARS-CoV-2 Spike peptides. In contrast, the hybrid group produced higher levels of innate immune associated cytokines IL-10 and IL-34, as well as the T cell homing chemokine CCL25, after stimulation.

**Conclusions:**

While both exposures generated comparable breadth of protection against SARS-CoV-2 variants, our findings suggest that the route of third exposure influences different aspects of the immune response, warranting further investigation into long-term immunity at both systemic and mucosal sites.

## Background

In 2021, COVID-19 vaccination campaigns were rapidly implemented worldwide, initially focusing on two-dose primary series using viral vectored or mRNA vaccines with an 8–12-week interval.^1,2^ These vaccines significantly reduced severe disease and mortality, particularly in high-risk groups.^3^ In the UK, vaccination schedules paused after the second dose, allowing health systems to evaluate immunity and transmission dynamics before widespread booster recommendations were made. Subsequent third exposures varied depending on individual circumstances and evolving viral epidemiology, being a mix of Delta or Omicron BA.1 infections. Additional mRNA vaccine doses were prioritized for individuals over 50 or with underlying health risks in the UK.^4^ This diverse exposure landscape, influenced by timing, viral variant, and vaccine policy, created a unique opportunity to compare immunological effects of vaccination or infection as third exposures.

We and others have demonstrated that a third vaccine dose provides significant additional protection against severe COVID-19 by increasing neutralizing antibody titres and boosting the T-cell response,^5–7^ and a prime-boost three dose schedule of COVID-19 vaccines is now considered optimal. However, the widespread rollout of booster doses presented challenges, particularly concerning cost and logistical burden of continued mass vaccination.^8^ This has led to ongoing discussions about the necessity of repeated immunizations, especially in populations with high rates of infection. With a large portion of the global population having been infected by SARS-CoV-2 at some point during the pandemic, it remains unclear whether infection offers comparable long-term protection to that provided by vaccination or if vaccination is still required for these individuals to maintain adequate immunity.

Our study leverages a natural comparison provided by the mass rollout of COVID-19 vaccines during ongoing infection transmission to investigate whether different types of immune exposure – here: 3 vaccines alone versus 2 vaccinations followed by an infection – result in differences in immune responses. Specifically, we aimed to compare detailed immune profiles (neutralizing antibody titres, pan immune cell profile, and functional T cell immune responses) between healthy adults receiving a booster dose as their third exposure and those whose third exposure was a SARS-CoV-2 infection.

## Methods

### Study design and Participants

We selected a sub-cohort of participants from the UCLH-Crick Legacy study (NCT04750356), a longitudinal study established in January 2021 by University College London Hospitals (UCLH) Biomedical Research Centre and the Francis Crick Institute, London, UK.^7^ Legacy was designed to track serological responses to COVID-19 vaccination during the national vaccination program in healthy staff volunteers. Participants underwent weekly PCR-based occupational health testing for SARS-CoV-2 infection during 2020–2022 as part of the UCLH-Crick PCR testing programme,^9^ ensuring comprehensive data on infection status throughout the study period. The study was approved by the London Camden and Kings Cross Health Research Authority Research Ethics Committee (IRAS number 286469) and was sponsored by University College London Hospitals.

**Fig. 1:**
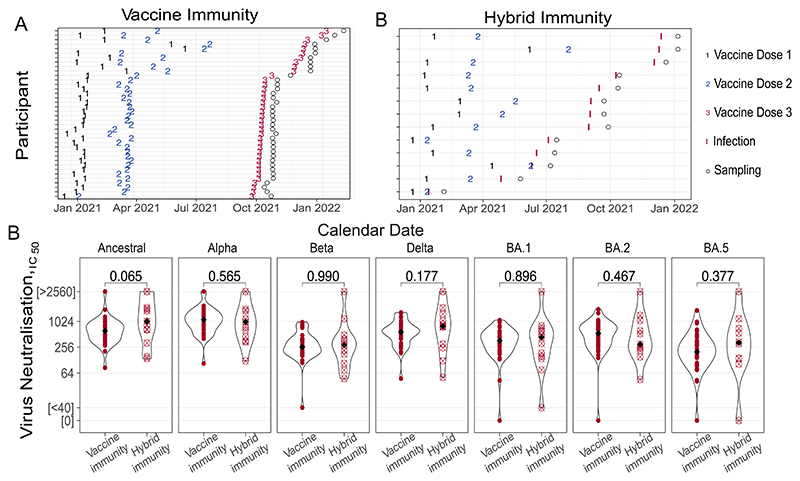
Neutralization titres after third exposure are comparable between vaccine and hybrid immunity. (A, B) Calendar dates of SARS-COV-2 exposure and sample collection following the third exposure for vaccine immunity group (A) and hybrid immunity group (B). (C) Neutralization titres against SARS-CO-2 variants, including the ancestral strain, Alpha, Beta, Delta and Omicron (BA.1, BA.2, BA.5), measured after the third exposure.

Third exposures were categorized as: (1) vaccine immunity: participants with no documented SARS-CoV-2 infection (negative anti-N IgG, negative weekly PCR, and no history of clinical COVID-19 episodes; *n* = 38); (2) hybrid immunity: participants with documented first positive SARS-CoV-2 PCR following two vaccine doses and prior negative anti-N IgG (*n* = 13). Overall participant demographics are provided in Supplementary Table 1, with assay-specific demographics in Supplementary Table 2. Vaccination and infection timelines are shown in Fig. 1A. Anti-SARS-COV-2 Nucleocapsid Roche assay was performed to confirm the absence of antibodies to the SARS-COV-2 Nucleocapsid (Supplementary Fig. 1).

### Neutralization assay

We quantified neutralising antibody titres (nAbT) against six SARS-CoV-2 variants including the ancestral strain, D614G, Alpha, Beta, Delta, and Omicron (BA.1, BA.2, and BA.5), using a high throughput microneutralisation assay at the COVID Surveillance Unit of the Francis Crick Institute as previously described.^7^ For details see Supplementary Appendix.

### Anti-nucleocapsid IgG detection

We measured anti-nucleocapsid IgG (anti-N IgG) in participant serum using the Elecsys Anti-SARS-COV-2 assay (Roche; 09203095190) run on the Cobas e411 analyser (Roche), in accordance with the manufacturer’s instructions. Results were reported as either reactive (positive) or non-reactive (negative).

### IFN-γ ELISpot assay

Cryopreserved PBMC were thawed, plated in pre-coated ELISpot plates from the Human IFN-γ ELISpotPRO kit (Mabtech 3420–2APT-10) and stimulated with SARS-CoV-2 peptide pools or controls. Details of peptide pools used are shown in Supplementary Table 3. Negative controls included unstimulated cells, while anti-CD3 was the positive control. Supernatants were saved for cytokine analysis. Plates were developed in a single step with human biotinylated IFN-γ detection Ab conjugated to alkaline phosphatase (7-B6–1-ALP, Mabtech). Results, expressed as IFN-γ spot-forming cells (SFC) per million PBMC, were adjusted by subtracting the background of unstimulated controls. Plates were read using the Mabtech ASTOR ELISpot reader.

### Luminex immunoassays

The pre-configured multiplex Human Immune Monitoring 80-plex ProcartaPlex Human Immune Response Panel Kit (EPX800–10080-901, Thermo Fisher) was used to measure 80 protein targets in cell culture supernatant on the Bio-Plex platform, using Luminex xMAP technology. All assays were conducted as per the manufacturer’s recommendations.

### CyTOF assay

PBMC were thawed in RPMI media supplemented with 20% sterile FCS and stained using the MaxPar Direct Immune Profiling Assay supplemented with T cell exhaustion markers (Standard BioTools, Supplementary Table 4) following the manufacturer’s instructions. Live/dead cells were identified using Cell-ID Intercalator-103Rh (Standard BioTools) and data acquired using CyTOF XT (Standard BioTools).

### T cell stimulation assay for flow cytometry

Thawed PBMC were stimulated for 6 h at 37 °C with either SARS-CoV-2 S-peptide pool (Supplementary Table 5), eBioscience stimulation cocktail as a positive control or no antigen as a negative control. Viability staining was done using Zombie Near Infrared dye followed by surface staining. Cells were then washed, fixed, permeabilized, stained for intracellular markers, and acquired on the Cytek Aurora. Gating strategy available in Supplementary Fig. 2 while list of antibodies used is shown in Supplementary Table 6.

### Data analysis

Study data were collected and managed using REDCap electronic data capture tools hosted at University College London. Metadata from REDCap was assembled and annotated in Chronogram package.^10^ Data from different machines were exported into R/FlowJo/GraphPad Prism/SPICE software for visualization and analysis. The statistical tests used are described below.

1. Viral neutralization
  Analysis was performed as previously described,^7,11^ with neutralizing antibody titers reported as IC50 values.
2. ELISPOT
  The number of SFC was compared between groups using the Wilcoxon rank-sum test.
3. Luminex
  We tested whether the effect of S pool stimulation on secreted protein concentrations differed between the two groups. A mixed effect model was used to test if analyte concentration was dependent on an interaction between groups and experimental condition (stimulated & unstimulated). The resulting nested models were compared with a likelihood ratio test to see if the inclusion of a particular interaction provided additional information with Benjamini-Hochberg (BH) multiple testing correction applied. The fold change was calculated for analytes that remained significantly different.
4. CyTOF assay
  Cleanup of FCS files was performed in FlowJo (Supplementary Fig. 3). The files were imported into R for analysis using the Spectre R package.^12^ This workflow included arcsinh transformation, clustering using FlowSOM,^13^ down sampling and dimensionality reduction using Uniform Manifold Approximation and Projection (UMAP).^14^ Statistical analysis was conducted using the diffcyt package.^15^ P-values were adjusted to account for multiple comparisons.
5. PBMC stimulation assay for flow cytometry

Data were analysed in FlowJo v10.9.0, (gating shown in Supplementary Fig. 2) and exported into GraphPad Prism v10.3.0 and SPICE software to generate figures, and R for statistical analysis. The Wilcoxon test with BH correction was applied to assess statistical differences between groups.

## Results

We identified 38 adults who were consistently negative for anti-N IgG, negative weekly PCR, and no history of clinical COVID-19 episodes in the vaccine-immunity cohort. We further identified 13 participants with a documented positive SARS-CoV-2 PCR following two vaccine doses and prior negative anti-N IgG in the hybrid-immunity cohort. All infections in the hybrid immunity group were mild and no participant required hospitalization.

The vaccine immunity group was older reflecting dose 3 national strategy, with a median age of 51 years [IQR: 34–59], compared to 33 years [IQR: 28–50] in the hybrid group. The proportion of female participants was similar between groups. The interval between the second and third exposures was longer in the vaccine group, with a median of 194 days [189 − 205] versus 130 days [97− 184] in the hybrid group. By contrast, the time from the third exposure to sampling was comparable, with medians of 19 days [16.2 – 20.8] and 24 days [[19 − 27]for the vaccine and hybrid groups, respectively (*p value = 0*.*196*). Detailed participant demographics are presented in Supplementary Table 1 and grouped by assay in Supplementary Table 2.

First, we compared the capacity of serum from both groups to neutralise various SARS-CoV-2 variants following a third SARS-CoV-2 exposure, accounting for time since exposures for vaccine (Fig. 1A) and hybrid immunity (Fig. 1B). Neutralizing antibody titres were comparable between cohorts irrespective of variant tested (Fig. 1C), suggesting both vaccine and hybrid immunity generate equivalently broad antibody titres against diverse viral strains.

**Fig. 2:**
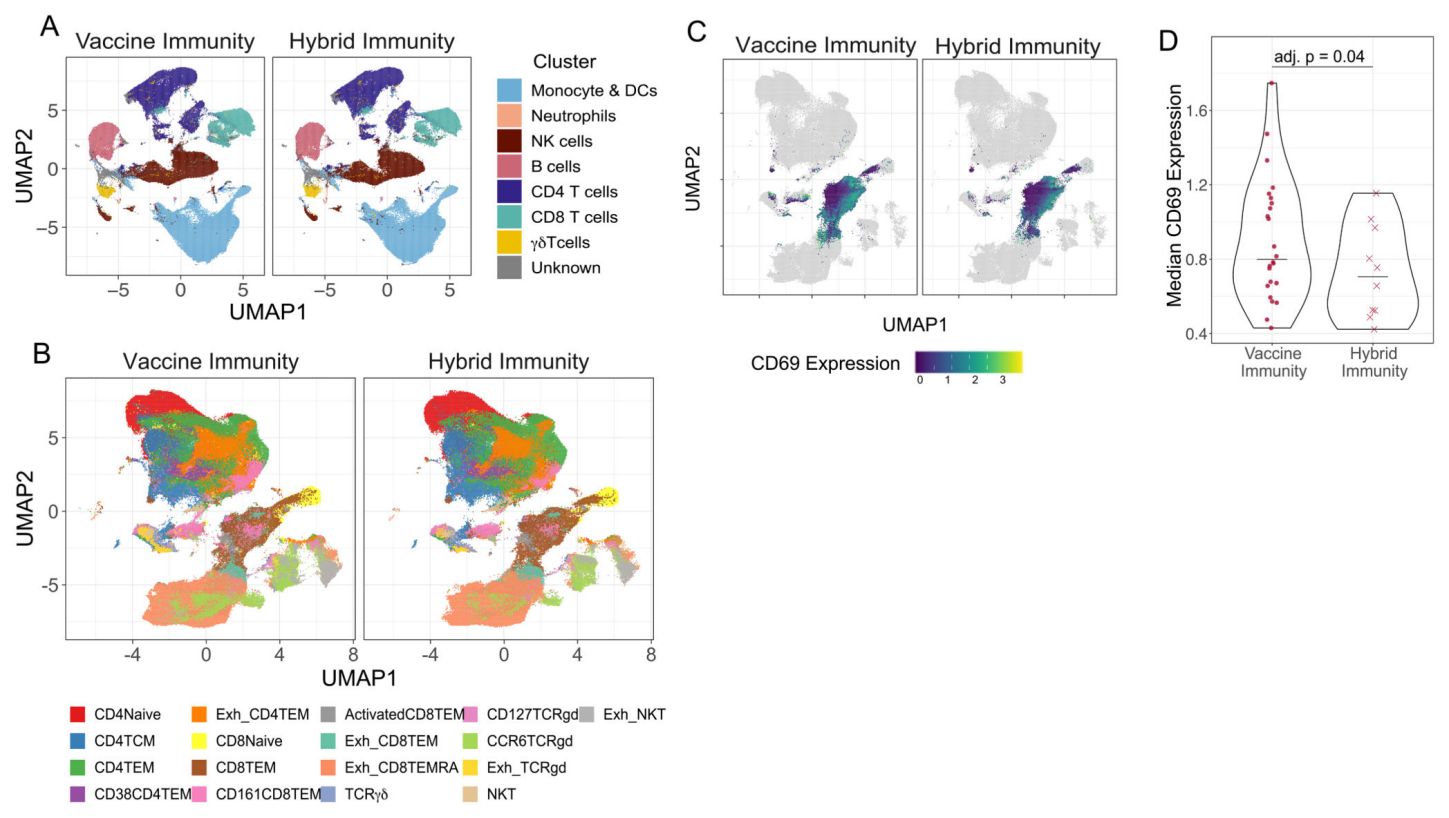
Circulating immune profiles are comparable after third exposures between vaccine-only and hybrid groups. (A) UMAP visualization of major immune cell lineages, including Monocytes & Dendritic cells, Neutrophils, NK cells, B cells, CD4 T cells, CD8 T cells, and γδ T cells, in individuals with vaccine and hybrid Immunity. (B) UMAP showing T cell subclusters stratified by vaccine and hybrid Immunity groups. (C) UMAP of T cell subclusters highlighting differences in CD69 expression within the CD8 TEM cluster between vaccine and hybrid Immunity groups. (D) Violin plot illustrating increased CD69 expression in the CD8 TEM cluster, as identified in (C).

Next, we compared cellular immunity between the two groups. Immune profiling of unstimulated PBMC was performed by CyTOF (antibody panel listed in Supplementary Table 4). We found similar abundance of clusters (Fig. 2A) and subclusters (Supplementary Fig. 4) of adaptive and innate immune cells, including B cells, CD4^+^, CD8^+^ and γδ T cells, NK, NKT, monocytes and dendritic cells. However, differential expression analysis indicated a significantly higher expression of the activation marker CD69 in the CD8^+^ T cell effector memory (TEM) cluster in the vaccine immunity group compared to the Hybrid group (Fig. 2C and D). Fig. 2B presents a UMAP of T cell subclusters, while Fig. 2C visualizes the same UMAP coloured by CD69 expression. A violin plot (Fig. 2D) further highlights the higher CD69 expression in the vaccine immunity group. These findings suggest that while overall immune cell composition remains similar, vaccination may drive subtle differences in T cell activation within effector memory CD8 T cells.

**Fig. 3:**
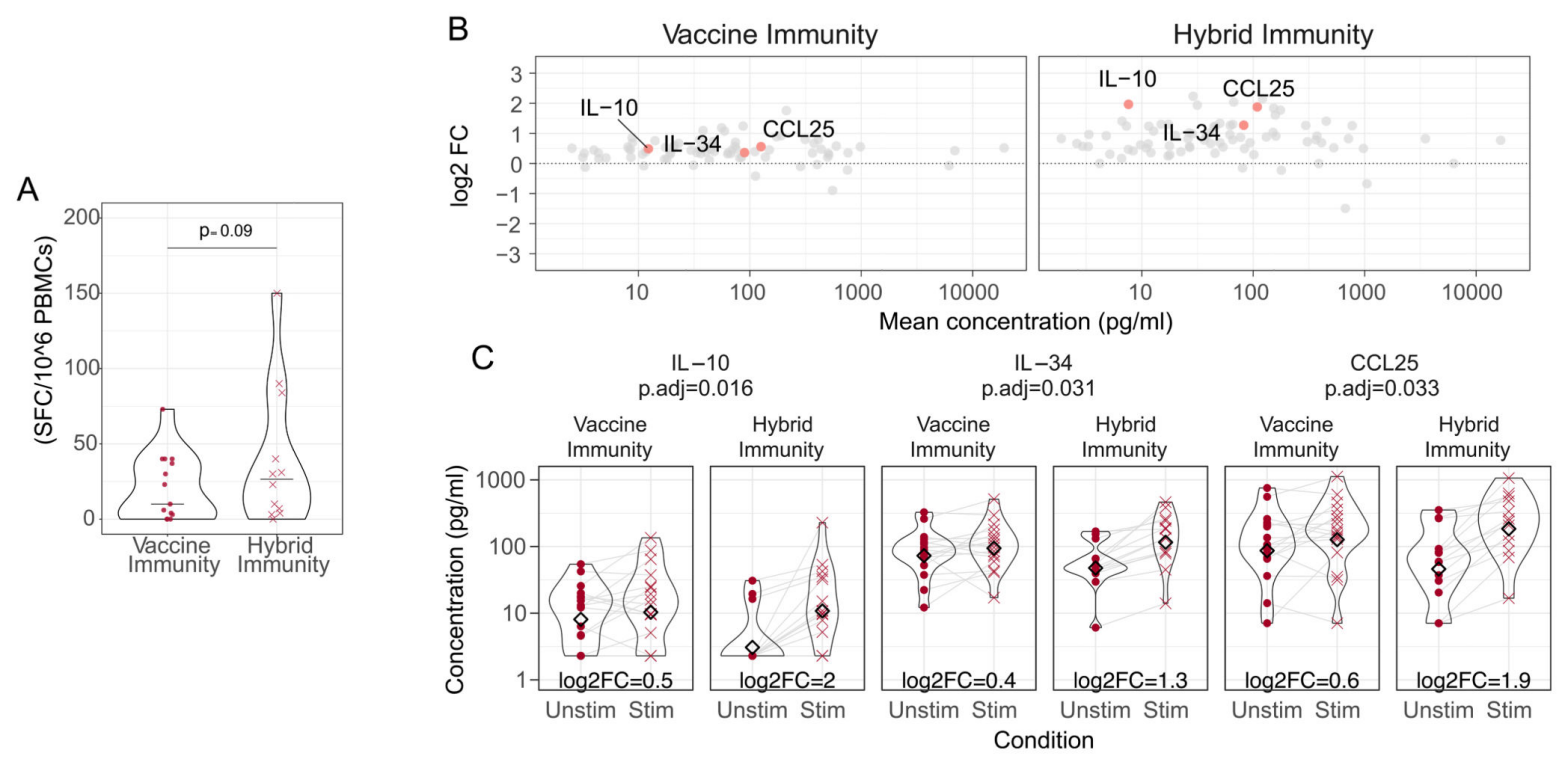
Higher production of IL-10, IL-34 and CCL25 in the hybrid immunity group upon stimulation with SARS-CoV-2 S antigen Pool. (A) Spot-forming cells (SFC) per million PBMC, comparing responses between the vaccine immunity and hybrid immunity groups. (B) MA plot showing the log fold change of analytes (Luminex assay) from baseline (0 on the y-axis) upon stimulation with the SARS-CoV-2 S antigen pool. The x-axis represents mean analyte concentration (pg/mL). Analytes highlighted in pink were statistically significant different between Vaccine and Hybrid immunity groups upon stimulation as shown in violin plots (C).

To evaluate broad antigen specific T cell responses between the groups, we used an in-house ELISpot assay to quantify production of IFN-γ by SARS-CoV-2-specific cells upon stimulation with a Spike peptide pool (Supplementary Table 3). The number of SFC per million PBMC, representing total IFN-γ T cell reactivity was comparable between groups (Fig. 3A). We next evaluated the in vitro production of 80 soluble analytes in the post-stimulation supernatant, using Luminex to better characterise and compare the antigen-specific cytokine response between the two groups. Following stimulation, the hybrid group exhibited a higher overall increase in production of soluble analytes compared to the vaccine group (Fig. 3B). Post-stimulation production of IL-10, IL-34 and CCL25 was significantly higher in the hybrid immunity group (Fig. 3C). These findings suggest a trend toward increased innate immune cell activation in the hybrid immunity group, potentially driven by elevated IL-34 production, while the rise in IL-10 may reflect a regulatory immune response. This is further supported by Supplementary Fig. 5, where other innate-related analytes (CSF3, GM-CSF, and CCL1) showed borderline significant elevations.

**Fig. 4:**
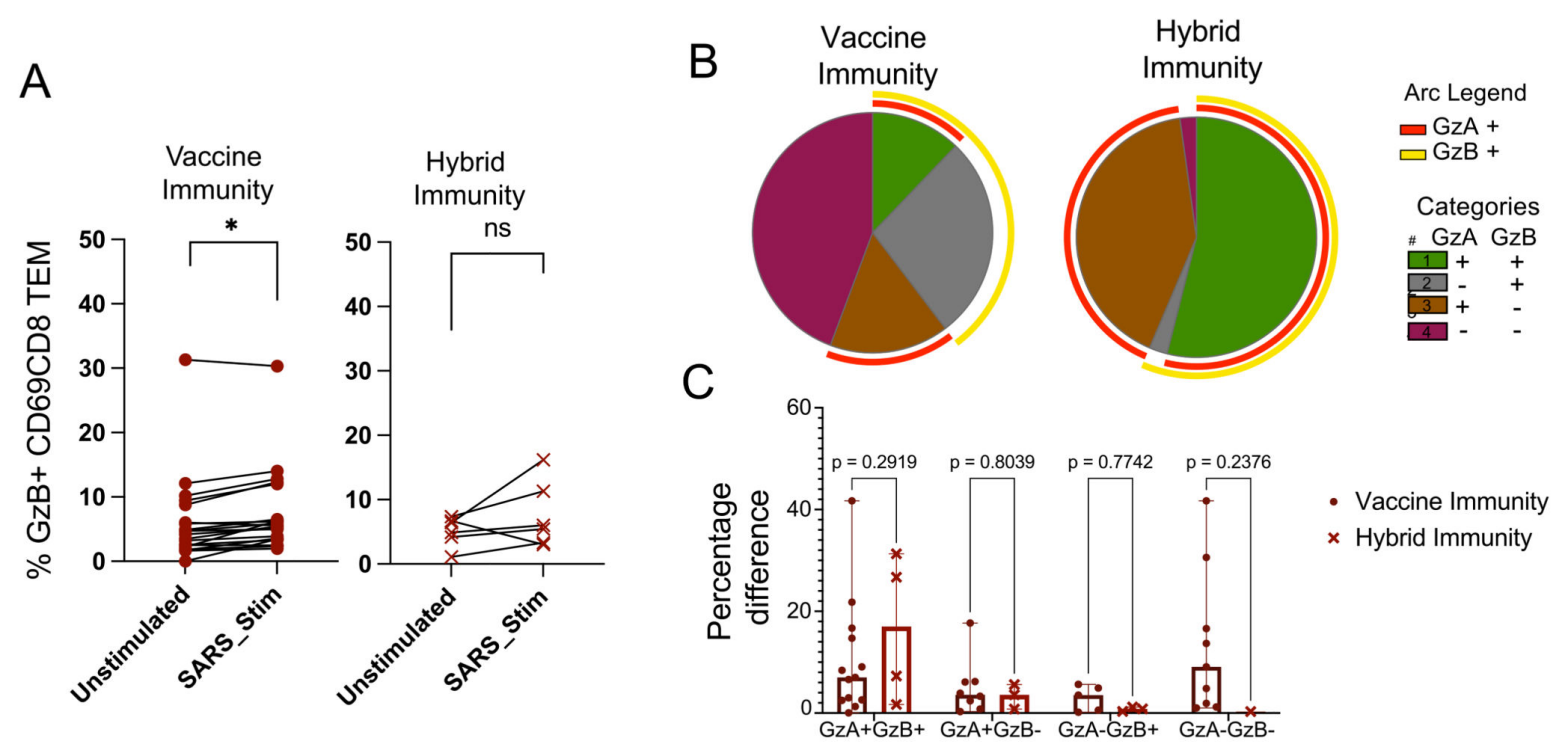
Higher Granzyme B production in vaccine immunity group following stimulation with SARS-CoV-2 S antigen Pool. (A) Changes in the proportion of Granzyme B (GzB)-positive cells among CD69+CD8TEM cells upon stimulation with S antigen pool for vaccine and hybrid immunity groups. (B, C) Polyfunctionality of CD69+CD8TEM cells based on Granzyme A and B expression. (B) Pie charts depict the distribution of polyfunctional subsets in vaccine and hybrid immunity groups. (C) Bar plots illustrate statistical significance between groups.

Given differences in the overall T cell cytokine response to the Spike peptide pool, we used intracellular cytokine staining in a subset of individuals with PBMC available (Supplementary Table 2), to further characterise Spike-specific T cells. Cells were stimulated with a SARS-CoV-2 Spike peptide pool (Supplementary Table 5) and analysed using a 28-color flow cytometry panel (Supplementary Table 6). We found increased expression of cytotoxic marker Granzyme B by CD69^+^ CD8^+^ TEM cells from the vaccine immunity group (Fig. 4A). We further assessed polyfunctionality of CD69^+^ CD8^+^ TEM cells based on intracellular Granzymes A and B. In the hybrid immunity group, most granzyme-positive cells co-expressed both Granzyme A and Granzyme B. In contrast, in the vaccine immunity group, a large proportion of CD69^+^ CD8^+^ TEM cells did not express either Granzyme (Fig. 4B). These differences did not reach the statistical significance threshold likely due to the limitation in PBMC sample availability (Fig. 4C).

## Discussion

Although exposure classification in large population studies is often limited by reliance on self-reported infection or vaccination status, extensive data have demonstrated that a third SARS-CoV-2 vaccine dose provides strong protective immunity against severe COVID-19.^16–19^ Third vaccine doses, given as part of a prime-boost schedule, enhance immunity by increasing neutralizing antibody titres and expanding antigen-specific B and T cell responses, even in immunocompromised individuals.^7,19–23^ However, by the time the third dose was introduced in late 2021, many individuals had been infected, raising the question of how a third exposure whether through infection or vaccination differentially shapes immunity after two prior vaccine doses. SARS-CoV-2 infection is frequently described to offer equal^24–26^ or greater^27,28^ protection compared to a vaccine, likely due to the form of exposure to antigens and induction of tissue specific memory, but reports are inconsistent. However, here we found that both types of third exposure elicited broadly comparable immune responses in blood.

Antibodies from both vaccine and hybrid immunity groups effectively neutralized multiple SARS-CoV-2 variants, including the divergent Omicron BA.1 and BA.5 as reported.^25,29,30^ While some studies reported higher neutralization in hybrid immunity groups,^31–34^ this may be explained by differences in disease severity, as most participants in our cohort had mild infections. These findings suggest that a third exposure, whether through vaccination or infection, induces similarly broad and effective antibodies against SARS-CoV-2 variants.

Our CyTOF analysis showed no evidence of immune dysregulation in the hybrid immunity group as previously reported.^35–37^ The frequency of both adaptive and innate immune cell subsets was comparable between groups. Interestingly, from the ELISPOT data, the number of S-specific cells were also similar between the two groups. *Urschel* et al., reported similar frequencies of S-specific cells between vaccine and hybrid even in the context of higher neutralization titres in the hybrid group.^34^ *Pušnik* et al., also showed that the S peptide specific B and T cells were not affected by different exposures.^38^ This data suggests that the SARS-CoV-2 S-specific responses are comparable between the two exposures in blood.

The vaccine immunity group exhibited higher expression of CD69 on CD8+ TEM cells. Notably, these cells were not SARS-CoV-2 specific, suggesting broad immune activation. This aligns with previous findings where CD69 expression was strongly induced in vaccinated individuals following stimulation with a superantigen indicating that vaccination may drive a heightened activation state independent of direct viral antigen exposure.^39^ Further analysis using intracellular cytokine staining following SARS-CoV-2 Spike peptide stimulation revealed higher Granzyme B expression within CD69^+^ CD8^+^ TEM cells in the vaccine immunity group which although modest, indicates that vaccine-induced CD8^+^ T cells are in a more activated state.

Overproduction of cytokines is a critical determinant of disease severity and mortality in SARS-CoV-2 infection.^40^ In our study, the hybrid immunity group exhibited increased production of IL-34, IL-10, and CCL25 upon stimulation. IL-34 is a monocyte-activating cytokine that may, in turn, contribute to the elevated levels of the anti-inflammatory cytokine IL-10. There was a trend toward innate activation as seen by high production of GM-CSF, CSF3 and CCL1. This suggests that prior infection may prime innate cells especially monocytes to modulate inflammation. The increased production of CCL25, a chemokine involved in T cell homing to mucosal tissues, further indicates a tissue-specific immune imprint from mucosal SARS-CoV-2 exposure.

While our study is the first to comprehensively compare the cellular and humoral immune response between these two groups, limitations include restricting analyses to peripheral blood (may not capture tissue specific responses, particularly in the hybrid immunity group); focusing on BA.1 infections may limit generalizability to other variants. Differences in age, timing of exposure, and sample collection between groups could also influence outcomes. Finally, whilst all the infection episodes within this sub-study were mild, with each SARS-CoV-2 infection there is a risk of persistent symptoms. Thus, whilst our data may suggest similar outcomes in systemic immunity from either exposure route, it could miss important differences in the severity or duration of symptoms after vaccination or infection.

Despite these limitations, our findings suggest that the route of third exposure influences different aspects of the immune response, warranting further investigation into long-term immunity at both systemic and mucosal sites.

## Supplementary Material

**Appendix A. Supporting information**

Supplementary data associated with this article can be found in the online version at doi:10.1016/j.jinf.2025.106598.

Supplementary Figure 1

Supplementary Figure 2

Supplementary Figure 3

Supplementary Figure 4

Supplementary Figure 5

Supplementary materials

## Data Availability

Data is available upon request to the Legacy study committee, which will confirm scientific validity of the query. Sharing of participant level data and metadata might require an MTA.

## Abbreviations

SARS-CoV-2: Severe Acute Respiratory Syndrome Coronavirus 2
COVID-19: Coronavirus Disease 2019
TEM: Effector Memory T cells
mRNA: messenger Ribonuclear
ELISpot: Enzyme-Linked ImmunoSpot
PCR: Polymerase Chain Reaction
AIM: Activation-Induced Marker
PBMC: Peripheral Blood Mononuclear Cells
ICS: Intracellular Cytokine Staining
