# Supplementary figures and images for: Third exposure to COVID-19 infection or vaccination differentially impacts T cell responses
Source: J Infect. Author manuscript; Available in PMC 2025 Nov 20. (PMC7618389; doi:10.1016/j.jinf.2025.106598)

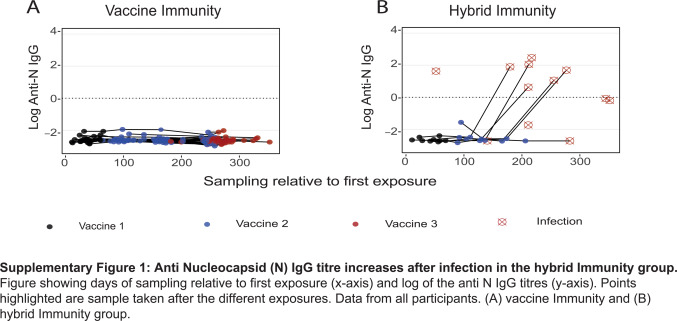

Supplement: Supplementary Figure 1 [file EMS210685-supplement-Supplementary_Figure_1.jpg]

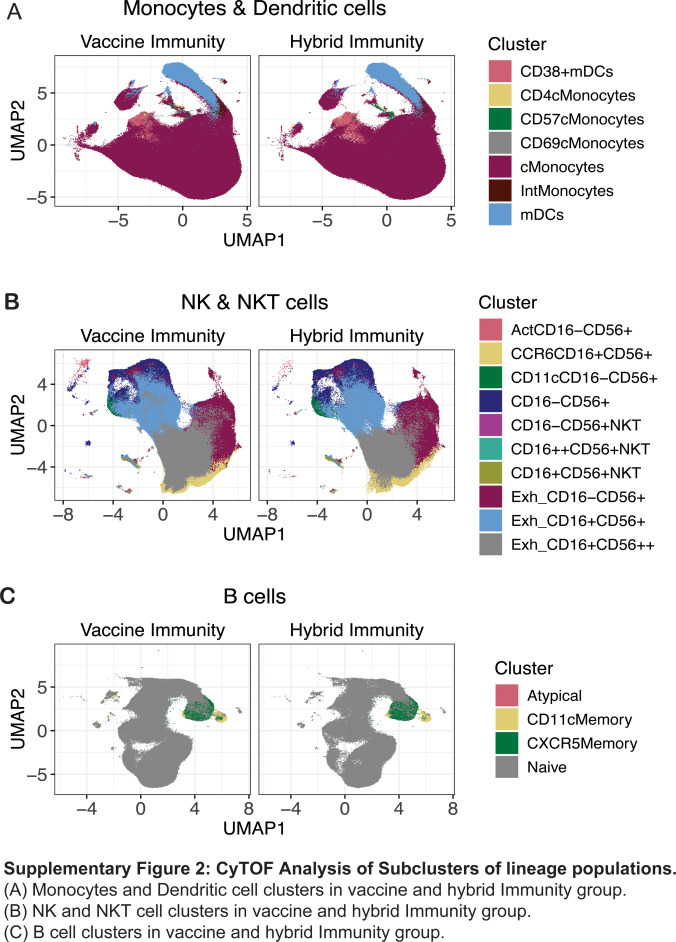

Supplement: Supplementary Figure 2 [file EMS210685-supplement-Supplementary_Figure_2.jpg]

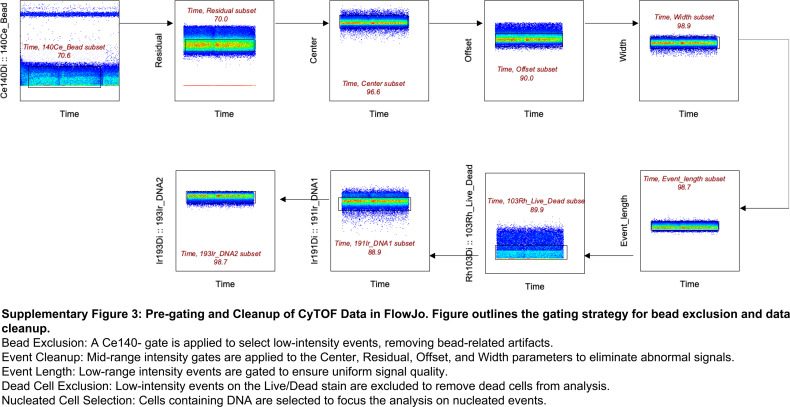

Supplement: Supplementary Figure 3 [file EMS210685-supplement-Supplementary_Figure_3.jpg]

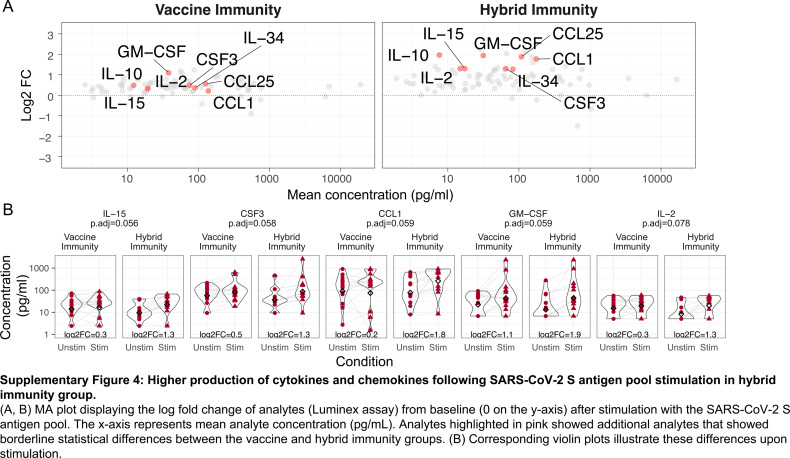

Supplement: Supplementary Figure 4 [file EMS210685-supplement-Supplementary_Figure_4.jpg]

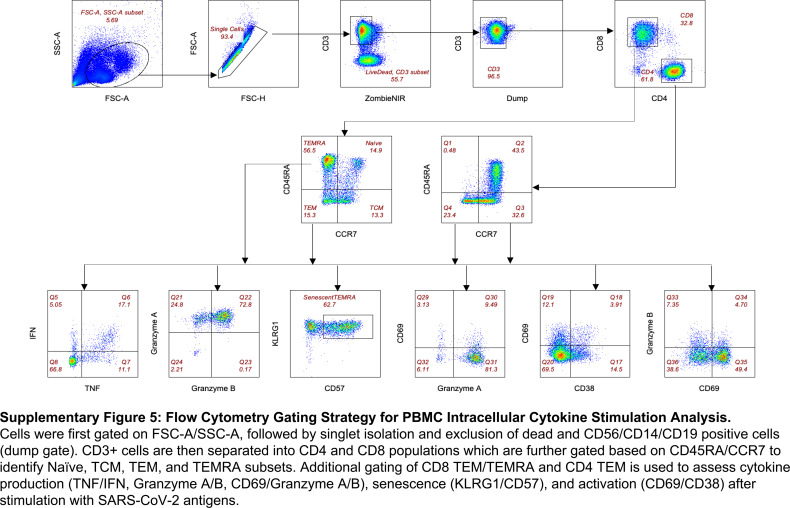

Supplement: Supplementary Figure 5 [file EMS210685-supplement-Supplementary_Figure_5.jpg]
