# Supplementary material for: Third exposure to COVID-19 infection or vaccination differentially impacts T cell responses
Source: J Infect. Author manuscript; Available in PMC 2025 Nov 20. (PMC7618389; doi:10.1016/j.jinf.2025.106598)
Supplement: Supplementary materials [file EMS210685-supplement-Supplementary_materials.pdf]

Third exposure to COVID-19 infection or vaccination differentially impacts T cell responses

Gift Ahimbisibwe[1], David Greenwood[1], Katalin Andrea Wilkinson[1,4], Joshua Gahir[1,5], Hermaleigh Townsley[1,5], Murad Miah[1], Philip Bawumia[1], Charlotte Chaloner[1,6], Dina Levi[1], Philip Hobson[1], Andy Riddell[1], Agnieszka Hobbs[2], Giulia Dowgier[2], Rebecca Penn[1], Theo Sanderson[1], Phoebe Stevenson-Leggett[2], Odiesia Daley[1], James Bazire[2], Ruth Harvey[3], Ashley S Fowler[1], Callie Smith[10], Mauro Miranda[1], Nicola O'Reilly[1], Scott Warchal[1], Karen Ambrose[1], Amy Strange[1], Gavin Kelly[1], Svend Kjar[1], *Legacy Investigators*, Bryan Williams[5,6], Vincenzo Libri[5,6], Steve Gamblin[1], Sonia Gandhi [1,6], Charles Swanton[1,6], David LV Bauer[1,7], Robert John Wilkinson[1,4,8], Edward J Carr[1,6], Emma C Wall*[1,5,9]

Table of Contents

Methods **2**

Neutralization assay *2*

IFN-gamma ELISpot assay *2*

Luminex Immunoassays *3*

CyTOF assay *3*

T cell stimulation assay *3*

References *4*

Supplementary tables **5**

Supplementary table legends *5*

Supplementary Table 1: Demographics of all included participants 6

Supplementary Table 2: Demographic details by assay 1

Supplementary Table 3: Luminex and ELISPOT SARS CoV-2 peptide pool 3

Supplementary Table 4: CyTOF panel 5

Supplementary Table 5: Flow cytometry Stimulation reagents 7

Supplementary Table 6: Flow cytometry Antibody panel 8

Supplementary Figures *9*

Methods

Neutralization assay

High-throughput live virus microneutralisation assays were performed as described previously[1,2]. Briefly, Vero E6 cells (Institute Pasteur) or Vero E6 cells expressing ACE2 and TMPRSS2 (VAT-1) (Centre for Virus Research) at 90-100% confluency in 384-well format were first titrated with varying MOIs of each SARS-CoV-2 variant and varying concentrations of a control monoclonal nanobody to normalise for possible replicative differences between variants and select conditions equivalent to wild-type virus. Following this calibration, cells were infected in the presence of serial dilutions of patient serum samples. After infection (24 hrs Vero E6 Pasteur, 16hrs VAT-1), cells were fixed with 4% final Formaldehyde, permeabilised with 0.2% TritonX-100, 3% BSA in PBS (v/v), and stained for SARS-CoV-2 N protein using Alexa488-labelled-CR3009 antibody produced in-house and cellular DNA using DAPI7. Whole-well imaging at 5x was carried out using an Opera Phenix (Perkin Elmer) and fluorescent areas and intensity calculated using the Phenix-associated software Harmony 9 (Perkin Elmer). Inhibition was estimated from the measured area of infected cells/total area occupied by all cells. The inhibitory profile of each serum sample was estimated by fitting a 4-parameter dose response curve executed in SciPy. Neutralising antibody titres are reported as the fold-dilution of serum required to inhibit 50% of viral replication (IC50), and are further annotated if they lie above the quantitative (complete inhibition) range, below the quantitative range but still within the qualitative range (i.e. partial inhibition is observed but a dose-response curve cannot be fit because it does not sufficiently span the IC50), or if they show no inhibition at all.

IFN-gamma ELISpot assay

Cryopreserved PBMCs were thawed with benzonase, then plated in pre-coated ELISpot plates and blocked with RPMI containing 10% FCS. Cells (300,000 per well) were stimulated with SARS-CoV-2 peptide pools or controls for 18–22 hours. These pools covered mutated and corresponding homologous WT spike regions. Details of SARS-CoV-2 peptide pools used are shown in Supplementary Table 3. Negative controls included unstimulated cells, while anti-CD3 was the positive control. After incubation, supernatants were saved for cytokine analysis. Plates were developed with a biotinylated IFN-γ antibody conjugated to alkaline phosphatase, followed by a phosphatase substrate. Results, expressed as IFN-γ spot-forming cells (SFC) per million PBMCs, were adjusted by subtracting the background from unstimulated controls. Plates were read using a Mabtech ASTOR ELISpot reader.

Luminex Immunoassays

The pre-configured multiplex Human Immune Monitoring 80-plex ProcartaPlex Human Immune Response Panel Kit (Invitrogen, Thermo Fisher Scientific cat number EPX800-10080-901) was used to measure a total of 80 protein targets in cell culture supernatant on the Bio-Plex platform (Bio-Rad Laboratories), using Luminex xMAP technology. All assays were conducted as per manufacturer recommendations. Protein concentrations were evaluated through fluorescence which is output as the mean fluorescence index (MFI). Negative controls containing no analyte were included to estimate the background noise. The MFI of the negative controls was subtracted from the MFI of the standard samples to control for inherent noise. Standards of known concentration for each analyte in the assay. A standard curve was generated for each analyte to estimate concentration in the samples. The minimum and maximum concentrations that can be reliably quantitated were called lower and higher limits of quantification (LLOQ and HLOQ), respectively. Values below the LLOQ and HLOQ therefore subject to censor.

CyTOF assay

PBMCs were thawed in RPMI media supplemented with 20% sterile-filtered fetal bovine serum (FBS) and stained using the MaxPar Direct Immune Profiling Assay (Fluidigm, now Standard BioTools) following the manufacturer's instructions. We supplemented the 30-panel antibody kit with the T cell expansion panel 3 to assess exhaustion (full panel details in Supplementary Table 4). Once stained and fixed, cells were stored at -80°C and processed in batches. Upon thawing, live/dead cell identification was conducted using Cell-ID Intercalator-103Rh (Standard BioTools) as per the manufacturer's protocol. Data acquisition was performed on a CyTOF XT (Standard BioTools), and collected events were bead-normalized using the platform's in-built algorithm.

T cell stimulation assay

Thawed PBMCs were stimulated with the SARS-CoV-2 S-peptide pool (Supplementary Table 5) at 37°C for 6 hours, with the eBioscience cell stimulation cocktail serving as a positive control and wells without antigen as negative controls. Following stimulation, cells were washed in RPMI media supplemented with 10% sterile-filtered fetal bovine serum (FBS) and blocked for 20 minutes using a cocktail of True-Stain Monocyte Blocker (BioLegend 426103), eBioscience Fc Receptor Binding Inhibitor Polyclonal Antibody (Thermo Fisher 14-9161-73), and FcR Blocking Reagent (Miltenyi 130-059-901) to prevent non-specific binding. Viability staining was performed using Zombie NIR Fixable Viability Dye

(BioLegend) for 20 minutes at 4°C in the dark. After additional washing, cells were resuspended in 100 µl of the surface antibody cocktail, incubated for 12 hours at room temperature, and then washed to remove excess antibody. Cells were subsequently fixed, permeabilized, and stained for intracellular markers before acquisition on the Cytek Aurora Spectral Analyzer. Compensation controls were applied to address spectral overlap, and Fluorescence Minus One (FMO) controls were used to establish gating thresholds. The data were analyzed using FlowJo software (version 10.9.0), with the gating strategy shown in Supplementary Figure 2 and antibodies listed in Supplementary Table 3.

References

1. Wall EC, Wu M, Harvey R, et al. Neutralising antibody activity against SARS-CoV-2 VOCs B.1.617.2 and B.1.351 by BNT162b2 vaccination. *The Lancet* 2021; 397(10292): 2331-3.

2. Wall EC, Wu M, Harvey R, et al. AZD1222-induced neutralising antibody activity against SARS-CoV-2 Delta VOC. *The Lancet* 2021; 398(10296): 207-9.

Supplementary tables

Supplementary table legends

Supplementary Table 1. Participant Demographics. Data shows collated demographics of all participants.
Supplementary Table 2. Assay-specific demographics. Data shows collated demographics of participants included in different assays: ELISPOT, live virus microneutralisation, Luminex assay, CyTOF and Intracellular Cytokine Staining.
Supplementary Table 3. Luminex and ELISPOT SARS CoV-2 S peptide pool
Supplementary Table 4. CyTOF antibody panel
Supplementary Table 5. Flow cytometry Stimulation reagents
Supplementary Table 6. Flow cytometry Antibody panel

Supplementary Table 1: Demographics of all included participants

Characteristic	Hybrid Immunity N = 13[1]	Vaccine Immunity N = 38[1]	p-value[2]
Median age (years) [IQR]	33 [28-50]	51 [34-59]	0.053
Sex			>0.9
Female	9 (69%)	26 (68%)	
Male	4 (31%)	12 (32%)	
First exposure			0.3
AZD1222	4 (31%)	5 (13%)	
BNT162b2	9 (69%)	31 (82%)	
mRNA1273		2 (5.3%)	
Second exposure			0.3
AZD1222	4 (31%)	5 (13%)	
BNT162b2	9 (69%)	31 (82%)	
mRNA1273		2 (5.3%)	
Third exposure			
Alpha	1 (7.7%)		
BA.1	2 (15%)		
Delta	8 (62%)		
Probable Alpha	1 (7.7%)		
Probable Delta	1 (7.7%)		
BNT162b2		38 (100%)	
Time from 2nd to 3rd Exposure (days) [IQR]	130 [97 - 184]	194 [189 – 205]	0.012[3]

Characteristic	Hybrid Immunity N = 13[1]	Vaccine Immunity N = 38[1]	p-value[2]
Time from 3rd Exposure to sampling (days) [IQR]	24 [19 – 27]	19 [16.2 – 20.8]	0.196[3]

[1]Median [25%-75%]; n (%)

[2]Wilcoxon rank sum test, Fisher’s exact test

[3]Wilcoxon rank sum test, Bonferroni

Supplementary Table 2: Demographic details by assay

		ELISPOT assay		Neutralisation assay		Luminex assay		CyTOF assay		Intracellular Cytokine Staining	
Metric		Vaccine Immunity (N = 16)[1]	Hybrid Immunity (N = 13)[1]	Vaccine Immunity (N = 38)[1]	Hybrid Immunity (N = 13)[1]	Vaccine Immunity (N = 15)[1]	Hybrid Immunity (N = 13)[1]	Vaccine Immunity (N = 37)[1]	Hybrid Immunity (N = 11)[1]	Vaccine Immunity (N = 20)[1]	Hybrid Immunity (N = 6)[1]
Age		57 [53-63]	33 [28-50]	51 [34-59]	33 [28-50]	56 [52-63]	33 [28-50]	51 [33-58]	33 [29-51]	50 [27-59]	31.0 [29.0-34.5]
Sex	Female	9 (56%)	9 (69%)	26 (68%)	9 (69%)	9 (60%)	9 (69%)	25 (68%)	7 (64%)	13 (65%)	4 (67%)
	Male	7 (44%)	4 (31%)	12 (32%)	4 (31%)	6 (40%)	4 (31%)	12 (32%)	4 (36%)	7 (35%)	2 (33%)
First exposure	AZD1222		4 (31%)	5 (13%)	4 (31%)		4 (31%)	5 (14%)	2 (18%)	4 (20%)	1 (17%)
	BNT162b2	16 (100%)	9 (69%)	31 (82%)	9 (69%)	15 (100%)	9 (69%)	30 (81%)	9 (82%)	14 (70%)	5 (83%)
	mRNA1273			2 (5.3%)				2 (5.4%)		2 (10%)	
Second exposure	AZD1222		4 (31%)	5 (13%)	4 (31%)		4 (31%)	5 (14%)	2 (18%)	4 (20%)	1 (17%)
	BNT162b2	16 (100%)	9 (69%)	31 (82%)	9 (69%)	15 (100%)	9 (69%)	30 (81%)	9 (82%)	14 (70%)	5 (83%)
	mRNA1273			2 (5.3%)				2 (5.4%)			2 (10%)
	Alpha		1 (7.7%)		1 (7.7%)		1 (7.7%)		1 (9.1%)		1 (17%)

		ELISPOT assay		Neutralisation assay		Luminex assay		CyTOF assay		Intracellular Cytokine Staining	
Metric		Vaccine Immunity (N = 16)[1]	Hybrid Immunity (N = 13)[1]	Vaccine Immunity (N = 38)[1]	Hybrid Immunity (N = 13)[1]	Vaccine Immunity (N = 15)[1]	Hybrid Immunity (N = 13)[1]	Vaccine Immunity (N = 37)[1]	Hybrid Immunity (N = 11)[1]	Vaccine Immunity (N = 20)[1]	Hybrid Immunity (N = 6)[1]
Third exposure	BA1		2 (15%)		2 (15%)		2 (15%)		2 (18%)		2 (33%)
	Delta		8 (62%)		8 (62%)		8 (62%)		6 (55%)		3 (50%)
	ProbAlpha		1 (7.7%)		1 (7.7%)		1 (7.7%)		1 (9.1%)		
	ProbDelta		1 (7.7%)		1 (7.7%)		1 (7.7%)		1 (9.1%)		
	BNT162b2	16 (100%)		38 (100%)		15 (100%)		37 (100%)		20 (100%)	

[1]Median [25%-75%]; n (%)

Supplementary Table 3: Luminex and ELISPOT SARS CoV-2 peptide pool

Reagent	Company	Cat.No
* PepTivator® SARS-CoV-2 Prot_S Complete	Miltenyi Biotech	130-127-041
*SARS-CoV-2 Prot_S B.1.1.529/BA.2 Mutation Pool	Miltenyi Biotech	130-130-807
* PepTivator® SARS-CoV-2 Prot_S B.1.1.529 (Omicron BA1)	Miltenyi Biotech	130-132-051
* PepTivator SARS-CoV-2 Prot_S B.1.617.2 Mutation pool (Delta)	Miltenyi Biotech	130-128-763
*PepTivator SARS-CoV-2 Prot_S1	Miltenyi Biotech	130-129-712
*PepTivator SARS-CoV-2 Prot_S	Miltenyi Biotech	130-126-701
*PepTivator SARS-CoV-2 Prot_M	Miltenyi Biotech	130-126-702
*PepTivator SARS-CoV-2 Prot_N	Miltenyi Biotech	130-126-698
*PepTivator SARS-CoV-2 Prot_S B.1.1.7 Mutation Pool (Alpha)	Miltenyi Biotech	130-127-844

*The SARS stimulant is provided as a 6 nmol/peptide lyophilized pool which was reconstituted by dissolving it in 200ul sterile filtered water to make a stock solution of PepTivator Peptides of 30 nmol (approximately 50 µg) of each peptide per ml. This is the stock from which the 20ul above is picked

Supplementary Table 4: CyTOF panel

	Marker	Clone	Metal
1.	CD45	HI30	89Y
2.	CD196 /CCR6	G034E3	141Pr
3.	CD123	6H6	143Nd
4.	CD19	HIB19	144Nd
5.	CD4	RPA-T4	145Nd
6.	CD8a	RPA-T8	146Nd
7.	CD11c	Bu15	147Sm
8.	CD16	3G8	148Nd
9.	CD45RO	UCHL1	149Sm
10.	CD45RA	HI100	150Nd
11.	CD161	HP-3G10	151Eu
12.	CD194/CCR4	L291H4	152Sm
13.	CD25	BC96	153Eu
14.	CD27	O323	154Sm
15.	CD57	HCD57	155Gd
16.	CD183/CXCR3	G025H7	156Gd
17.	CD185/CXCR5	J252D4	158Gd
18.	CD28	CD28.2	160Gd
19.	CD38	HB-7	161Dy
20.	CD56/NCAM	NCAM16.2	163Dy
21.	TCRγδ	B1	164Dy
22.	CD294	BM16	166Er
23.	CD197/CCR7	G043H7	167Er
24.	CD14	63D3	168Er
25.	CD3	UCHT1	170Er
26.	CD20	2H7	171Yb
27.	CD66b	G10F5	172Yb
28.	HLA-DR	LN3	173Yb
29.	IgD	IA6-2	174Yb
30.	CD127	A019D5	176Yb
Extra antibodies not part of the lyophilized cocktail			
31.	OX40/CD134		142Nd
32.	TIGIT		159Tb

33.	TIM3/CD366		169Tm
34.	ICOS/CD278		175Lu
35.	CD137		209Bi
36.	CD69		162Dy
37.	PD-1/CD279		165Ho
38.	Cell-ID Intercalator-103Rh		103Rh

Supplementary Table 5: Flow cytometry Stimulation reagents

Reagent	Company	Cat.No
SARS-CoV-2 antigen stimulation pool		
*SARS-CoV-2 Prot_S+	Miltenyi Biotech	130-127-311
*SARS-CoV-2 MHC-I Select Prot_S	Miltenyi Biotech	130-130-634
*SARS-CoV-2 Prot_S B.1.1.529/BA.2 Mutation Pool	Miltenyi Biotech	130-130-807
*SARS-CoV-2 Prot_S B.1.1.529/BA.5 Mutation Pool	Miltenyi Biotech	130-132-051
*SARS-CoV-2 Prot_S B.1.617.2 Mutation Pool (Delta)	Miltenyi Biotech	130-128-761
*SARS-CoV-2 Prot_S B.1.1.7 Mutation Pool (Alpha)	Miltenyi Biotech	130-127-844
Cell Stimulation Cocktail (500x)	eBioscience	00-4970-93
CD28/CD49d (1000x)	BD FastImmune™	347690
*The SARS stimulant is provided as a 6 nmol/peptide lyophilized pool which was reconstituted by dissolving it in 200ul sterile filtered water to make a stock solution of PepTivator Peptides of 30 nmol (approximately 50 µg) of each peptide per ml. This is the stock from which the 20ul above is picked		

Supplementary Table 6: Flow cytometry Antibody panel

	Reagent	Company	Dilution(ul/100ul)	Cat.No
1.	CD8 SBV475	BioRad	*0.025*	MCA1226SBV475
2.	CD27 Monoclonal Antibody (LG.7F9), APC-eFluor™ 780	eBiosciences	*0.07*	47-0271-80
3.	CD38 PE-Fire810	Sony	*0.1*	2586125
4.	PD-1 PE-Cy7	BioLegend	*0.1*	329918
5.	CD4 SBB480	BioRad	*0.2*	MCA1267SBB580
6.	HLA-DR APC-Fire810	BioLegend	*0.2*	307674
7.	CCR6 BV786	BD	*0.2*	563704
8.	TIGIT BUV615	BD	*0.2*	570374
9.	CD56 BV510	BioLegend	*0.25*	318340
10.	CD19 BV510	BioLegend	*0.25*	302242
11.	CD14 BV510	BioLegend	*0.25*	301842
12.	Hu CD107a RB545 H4A3	BD	*0.25*	569765
13.	CD127 Monoclonal Antibody (eBioRDR5)	LifeTechnologies	*0.25*	365-1278-41
14.	TIM-3 PE-Cy5	BioLegend	*0.4*	345052
15.	Spark YG 581 anti-human CD197 (CCR7)	BioLegend	*0.4*	353265
16.	BV711 anti-human CD69	BioLegend	*0.4*	310943
17.	Hu KLRG1 R718 Z7-205	BD	*0.4*	568660

18.	CD39 BUV496	BD	*0.5*	749969
19.	CD57 BV605	BioLegend	*0.5*	393304
20.	CD28 BUV737	BD	*0.6*	748475
21.	Brilliant Violet 570 anti-human CD3 Antibody	BioLegend	*1*	300436
22.	CD45RA SBB810	BioRad	*1.5*	MCA88SBB810
23.	Alexa Fluor® 647 anti-human Granzyme A	BioLegend	*0.2*	507214
24.	TNF RB705	BD Horizon	*0.25*	570621
25.	PE/Dazzle 594 anti-human/mouse Granzyme B	BioLegend	*0.3*	372215
26.	IFN gamma Mouse anti-human BUV661	Life Technologies	*0.5*	2856306
27.	IL-17A Monoclonal Antibody (eBio64DEC17) FITC	eBiosciences	*1*	11-719-42
28.	Brilliant Violet 650 anti-human IL-2	BioLegend	*2.2*	500333

Supplementary Figures

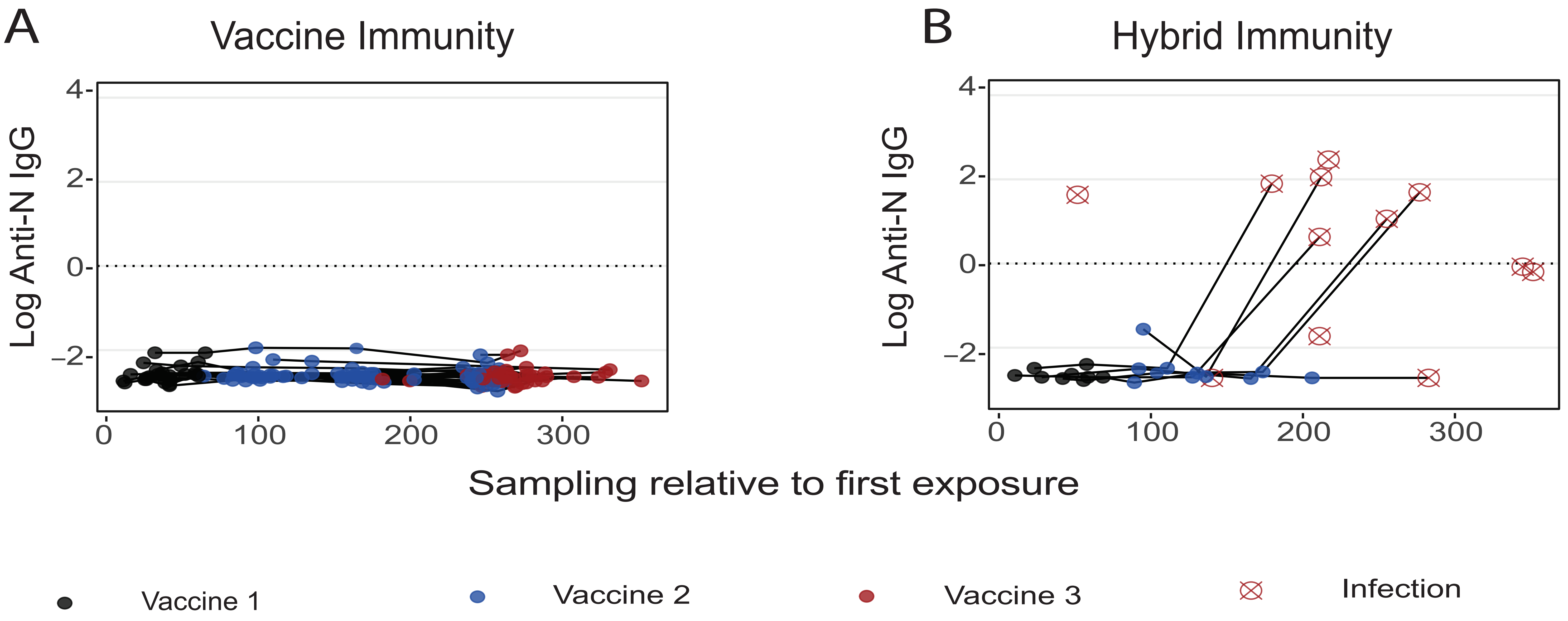

Supplementary Figure 1: Anti Neucleocapsid (N) IgG titre increases following infection in the Hybrid Immunity group

Figure showing days of sampling relative to first exposure (x-axis) and log of the anti N IgG titres (y-axis). Points highlighted are sample as taken after the different exposures. Data from all participants. (A) Vaccine Immunity and (B) Hybrid Immunity group.

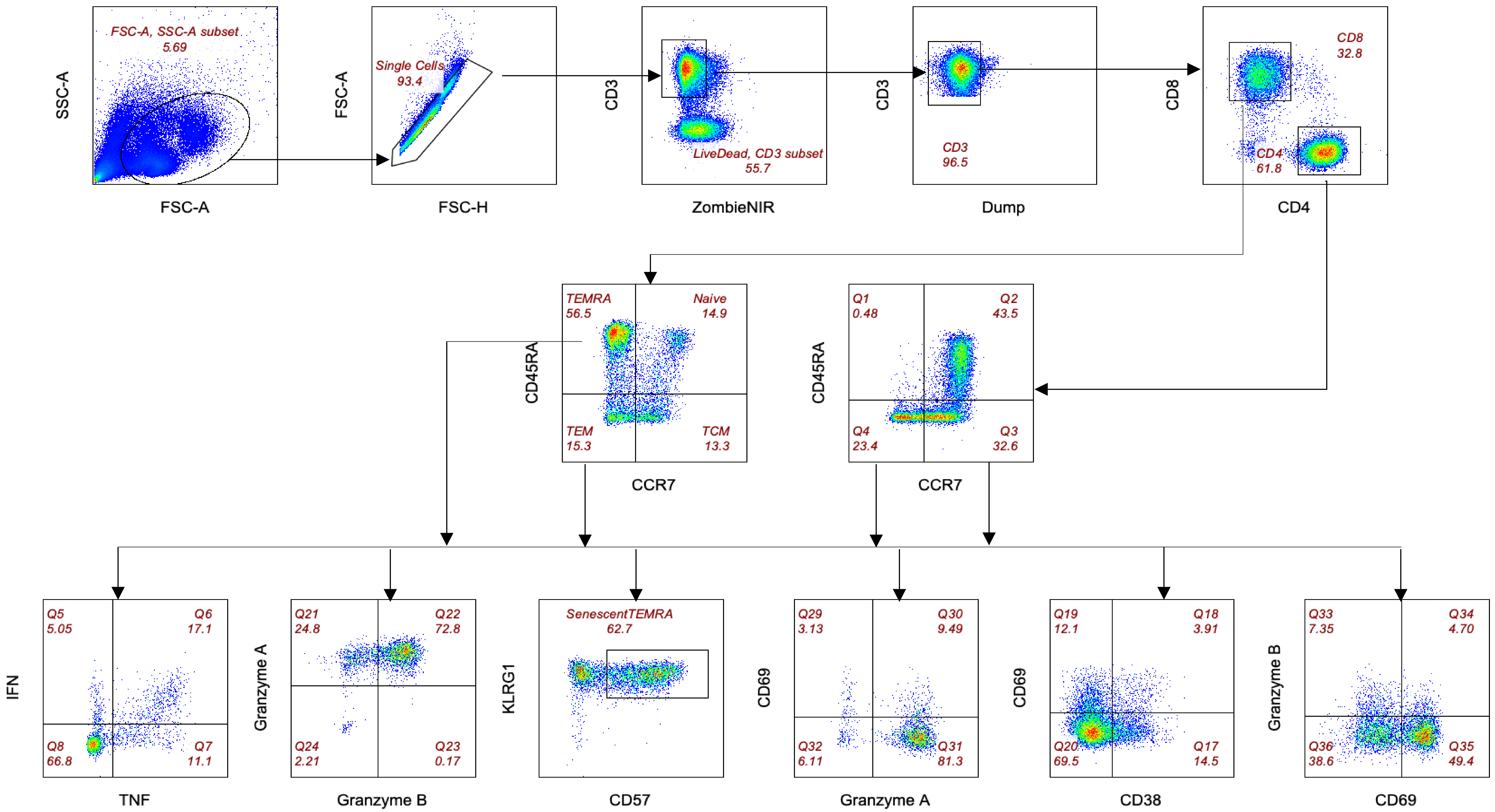

Supplementary Figure 2: Flow Cytometry Gating Strategy for PBMC Intracellular Cytokine Stimulation Analysis.
Cells were first gated on FSC-A/SSC-A, followed by singlet isolation and exclusion of dead and CD56/CD14/CD19 positive cells (dump gate).
CD3+ cells are then separated into CD4 and CD8 populations which are further gated based on CD45RA/CCR7 to identify Naïve, TCM, TEM, and TEMRA subsets.
Additional gating of CD8 TEM/TEMRA and CD4 TEM is used to assess cytokine production (TNF/IFN, Granzyme A/B, CD69/Granzyme A/B), senescence (KLRG1/CD57), and activation (CD69/CD38) after stimulation with SARS-CoV-2 antigens.

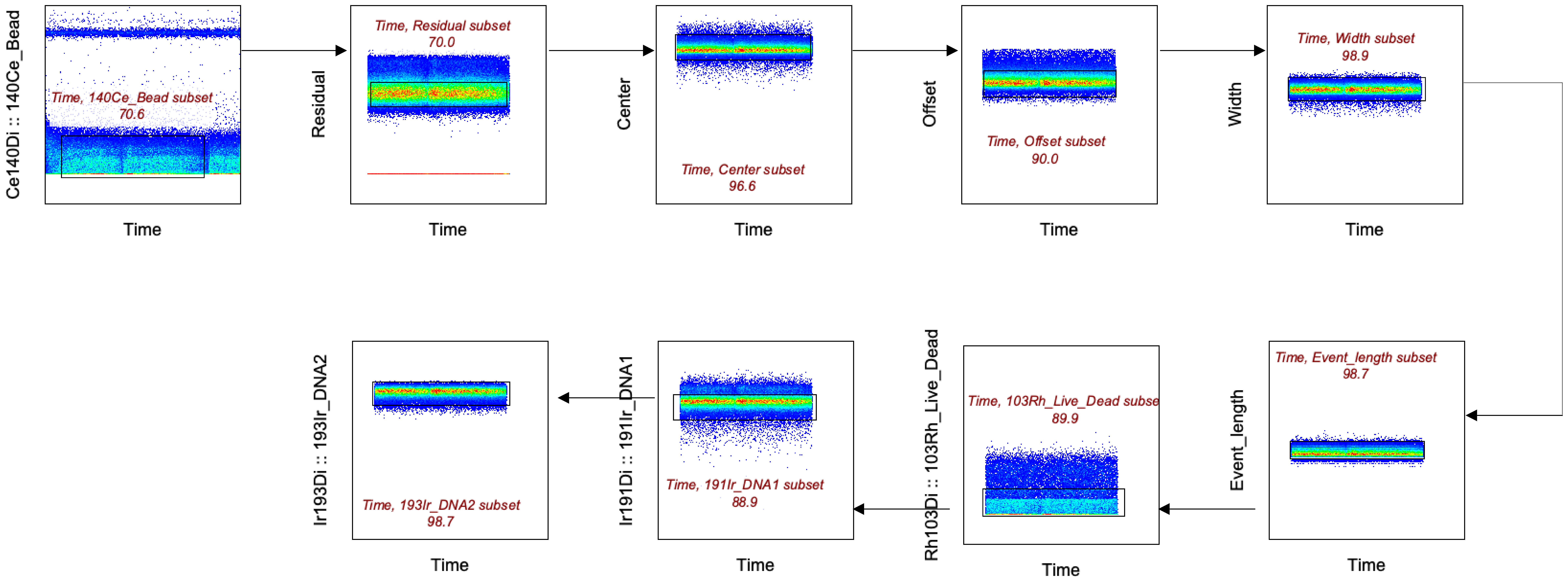

Supplementary Figure 3: Pre-gating and Cleanup of CyTOF Data in FlowJo. Figure outlines the gating strategy for bead exclusion and data cleanup.

- Bead Exclusion: A Ce140- gate is applied to select low-intensity events, removing bead-related artifacts.
- Event Cleanup: Mid-range intensity gates are applied to the Center, Residual, Offset, and Width parameters to eliminate abnormal signals.
- Event Length: Low-range intensity events are gated to ensure uniform signal quality.
- Dead Cell Exclusion: Low-intensity events on the Live/Dead stain are excluded to remove dead cells from analysis.
- Nucleated Cell Selection: Cells containing DNA are selected to focus the analysis on nucleated events.

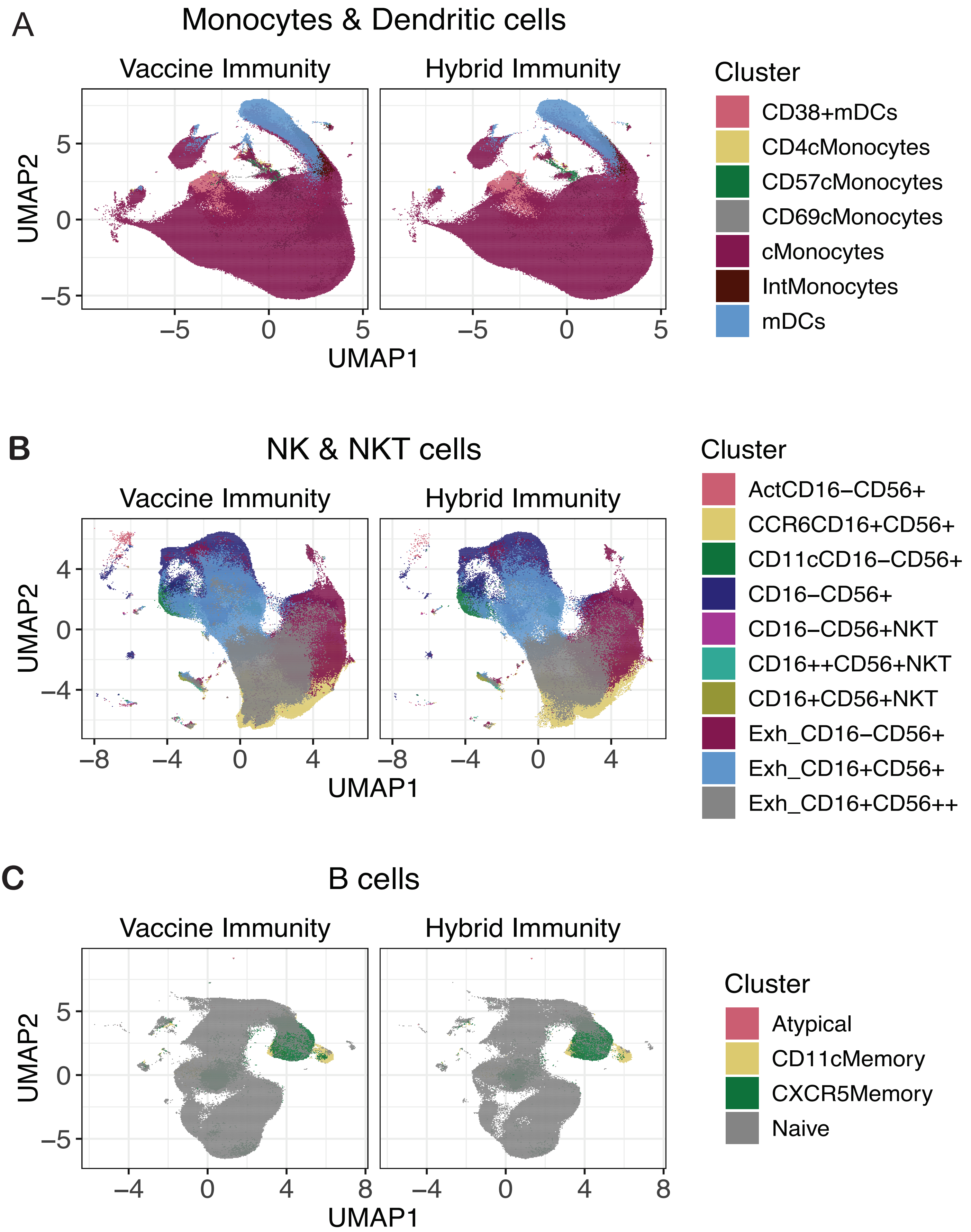

Supplementary Figure 4: CyTOF Analysis of Subclusters of lineage populations

(A) Monocytes and Dendrtic cell clusters in Vaccine and Hybrid Immunity group

(B) NK and NKT cell clusters in Vaccine and Hybrid Immunity group

(C) B cell clusters in Vaccine and Hybrid Immunity group

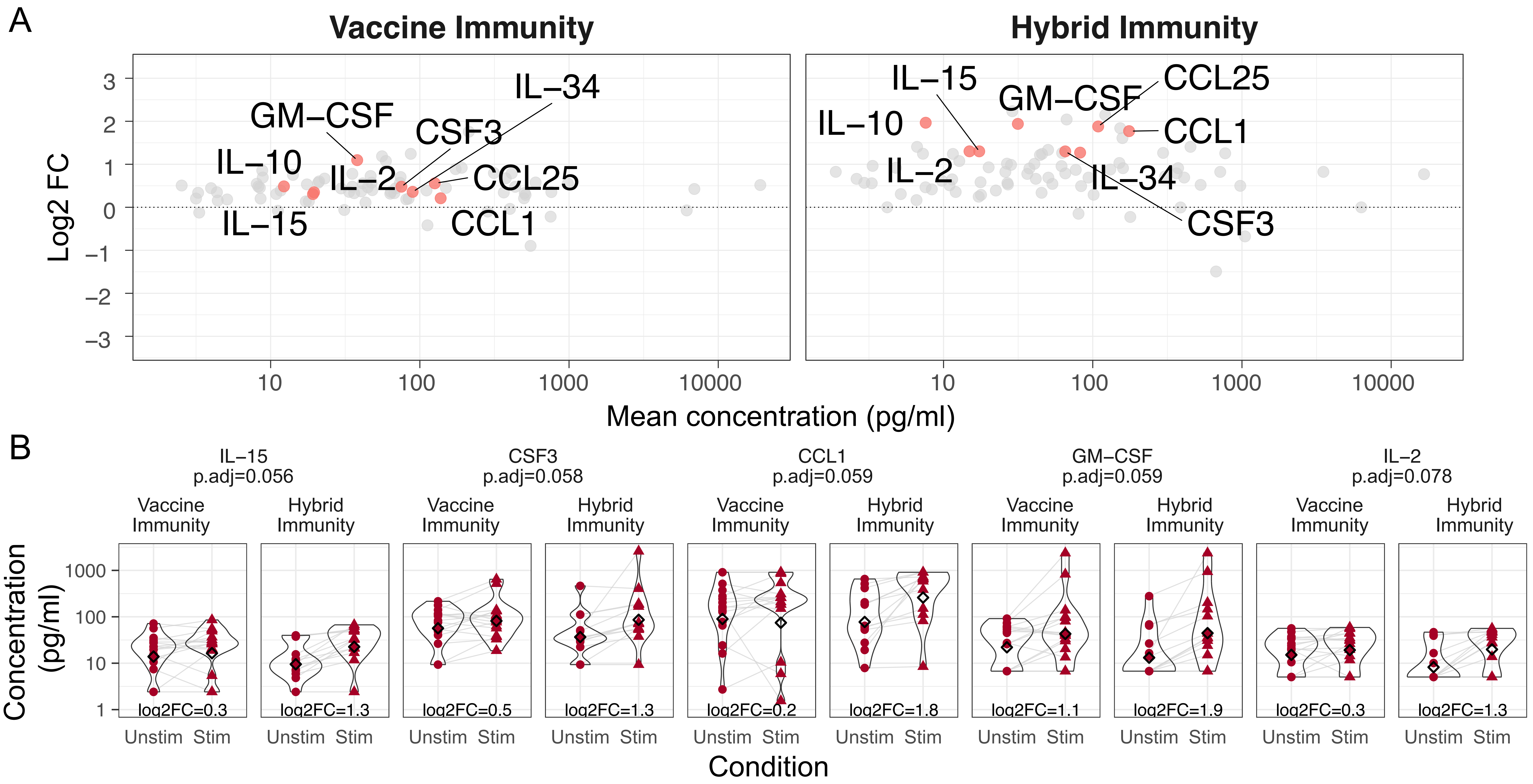

Supplementary Figure 5: Higher production of cytokines and chemokines following SARS-CoV-2 S antigen pool stimulation in Hybrid immunity group

(A, B) MA plot displaying the log fold change of analytes (Luminex assay) from baseline (0 on the y-axis) after stimulation with the SARS-CoV-2 S antigen pool. The x-axis represents mean analyte concentration (pg/mL). Analytes highlighted in pink showed statistically significant and borderline differences between the vaccine and hybrid immunity groups. (B) Corresponding violin plots illustrate these differences upon stimulation. p.adj refers to adjusted p-values after multiple testing correction.
